# PMVT: a lightweight vision transformer for plant disease identification on mobile devices

**DOI:** 10.3389/fpls.2023.1256773

**Published:** 2023-09-26

**Authors:** Guoqiang Li, Yuchao Wang, Qing Zhao, Peiyan Yuan, Baofang Chang

**Affiliations:** ^1^ Institute of Agricultural Economics and Information, Henan Academy of Agricultural Sciences, Zhengzhou, Henan, China; ^2^ College of Computer and Information Engineering, Henan Normal University, Xinxiang, Henan, China; ^3^ Key Laboratory of Artificial Intelligence and Personalized Learning in Education of Henan Province, Xinxiang, Henan, China

**Keywords:** plant disease identification, vision transformer, lightweight model, attention module, APP

## Abstract

Due to the constraints of agricultural computing resources and the diversity of plant diseases, it is challenging to achieve the desired accuracy rate while keeping the network lightweight. In this paper, we proposed a computationally efficient deep learning architecture based on the mobile vision transformer (MobileViT) for real-time detection of plant diseases, which we called plant-based MobileViT (PMVT). Our proposed model was designed to be highly accurate and low-cost, making it suitable for deployment on mobile devices with limited resources. Specifically, we replaced the convolution block in MobileViT with an inverted residual structure that employs a 7×7 convolution kernel to effectively model long-distance dependencies between different leaves in plant disease images. Furthermore, inspired by the concept of multi-level attention in computer vision tasks, we integrated a convolutional block attention module (CBAM) into the standard ViT encoder. This integration allows the network to effectively avoid irrelevant information and focus on essential features. The PMVT network achieves reduced parameter counts compared to alternative networks on various mobile devices while maintaining high accuracy across different vision tasks. Extensive experiments on multiple agricultural datasets, including wheat, coffee, and rice, demonstrate that the proposed method outperforms the current best lightweight and heavyweight models. On the wheat dataset, PMVT achieves the highest accuracy of 93.6% using approximately 0.98 million (M) parameters. This accuracy is 1.6% higher than that of MobileNetV3. Under the same parameters, PMVT achieved an accuracy of 85.4% on the coffee dataset, surpassing SqueezeNet by 2.3%. Furthermore, out method achieved an accuracy of 93.1% on the rice dataset, surpassing MobileNetV3 by 3.4%. Additionally, we developed a plant disease diagnosis app and successfully used the trained PMVT model to identify plant disease in different scenarios.

## 1 Introduction

Plant disease is one of the contributing factors to the global decrease in grain production (Savary et al., 2019), and real-time detection of plant disease has an important impact on the agricultural industry. Applying deep learning models significantly simplifies the entire process and enables end-to-end technical services. Currently, there are two typical architectures for plant disease recognition: convolutional neural network (CNN)-based architectures and vision transformer (ViT)-based architectures. These methods extract explicit features from images and automatically perform classification, which is key for plant disease recognition.

Over the past few years, the application of CNNs to identifying plant diseases has gained in popularity with the development of artificial intelligence technology. For instance, Akshai and Anitha (2021) compared various CNNs using the PlantVillage dataset (Hughes and Salathe 2015) and reported that the DenseNet model with feature map reuse achieved the highest accuracy of 98.27%. Another study by Yu et al. (2022a) used a ResNet network with a residual structure to identify apple leaf diseases, and it obtained an average F1-score of 95.70%. CNNs can efficiently extract significant features from images and accomplish plant disease identification automatically. The primary reason for this is that CNNs have the characteristic of parameter sharing, which reduces the number of parameters in the model and addresses the overfitting issue seen in computer vision tasks. Therefore, the application of deep learning technology based on CNNs has made significant progress in plant disease diagnosis (Hasan et al., 2020; Xiong et al., 2021; Ahmad et al., 2022). Nonetheless, there will be an increase in unnecessary computational overhead as a network’s depth increases. Simultaneously, the convolutional layer of CNNs only considers the characteristics of the local area during convolution and does not explicitly incorporate the positional information of pixels. This will impact the effectiveness of a plant disease identification model.

To address the above issues, Dosovitskiy et al. (2020) proposed a vision transformer (ViT) architecture based on a self-attention mechanism (Vaswani et al., 2017) to replace the traditional CNN for image recognition. A ViT architecture divides an image into non-overlapping patches and applies multi-head self-attention within the transformer encoder to learn representations of patches. Although this paradigm considers the global relationship of images and has achieved satisfactory results in plant disease recognition, it usually requires a large quantity of training data to achieve relatively high accuracy. Hence, alternating the use of CNNs and ViTs to extract more comprehensive features has become a better choice in plant disease diagnosis. Take a classic case: Lu et al. (2022) introduced a ghost module into the ViT encoder, which extracts different levels of features in an image. Their model achieved an accuracy rate of 98.14% in detecting grape leaf diseases and insect pests in the field. Similarly, Yu et al. (2023) used inception blocks to enhance the ability of the ViT encoder to extract local information; they achieved optimal performance on four typical plant disease datasets. As an alternative architectural paradigm to CNNs, the ViT has attracted significant attention and achieved considerable success in the field of computer vision (Khan et al., 2022; Lin et al., 2022).

With the significant advancements of CNNs and ViT networks in plant disease recognition technology, a prevailing trend among network models is to augment the number of parameters in order to enhance performance. These enhancements in performance are accompanied by an increase in model size (network parameters) and latency (Han et al., 2021; Wu et al., 2021; Yu et al., 2022b). They overlook a common issue: plant disease identification is typically conducted on edge devices, such as smartphones and embedded devices. Such devices usually have restricted computing power, storage capacity, and energy supply. Hence, using a lightweight network can decrease the size and computational complexity of the model, thereby improving its compatibility with resource constraints. Numerous researchers have recently been studying the application of affordable network models for real-time plant disease detection. Concretely, Bao et al. (2021) proposed SimpleNet, which achieved 94.10% wheat recognition accuracy with only 2.13 million (M) parameters. In addition, the apple leaf disease identification method based on the cascade backbone network (CBNet) proposed by (Sheng et al., 2022) achieved an accuracy rate of 96.76%. Moreover, the VGG-ICNN model proposed by Thakur et al. (2023) has 6 M parameters, which is lower than most deep learning models; and it performs well on multiple datasets such as apple, corn, and rice. Generally, the methods mentioned above primarily concentrate on identifying a single plant disease, while other methods exhibit imbalances in identification accuracy and calculation cost. Hence, to enhance the real-time performance of plant disease identification, it is crucial to employ a low-latency and highly accurate network model.

Achieving high-accuracy and low-cost plant disease identification in agricultural environments with limited computing resources presents a significant challenge. The majority of existing lightweight networks focus on a single plant disease. However, when faced with numerous types of plant diseases, they fail to deliver satisfactory performance. In this paper, we introduced a lightweight model for plant disease diagnosis based on MobileViT (Mehta and Rastegari, 2021), which has a low computational cost and is competitive in terms of inference speed. In particular, the crisscrossing leaves in the agricultural dataset lead to an unsatisfactory recognition effect with MobileViT. Thus, we consider using a larger convolution kernel (7 × 7) to analyze the connection between different leaves. Using larger convolution kernels allows us to model the dependencies between long-distance pixels (Liu et al., 2021; Liu et al., 2022) and enhance the ability of the model to capture global information from plant disease images. Additionally, focusing on the salient leaf regions in plant images can improve the robustness of the model. We used the CBAM (Woo et al., 2018) to adjust feature weights in various channels of the transformer encoder. Finally, we employed a residual network to fuse the initial feature map and improve the fitting ability of the model. We named this model plant-based MobileViT (PMVT) and deployed it to identify plant diseases in datasets and in various scenarios. Experimental results indicate that PMVT surpasses the current leading lightweight networks and heavyweight models, thereby demonstrating its effectiveness as a versatile backbone network across various datasets.

The main contributions of this paper are as follows.

- We used a low-cost ViT model for plant disease diagnosis. This model is computationally efficient and can function as a generic backbone network on mobile devices.
- We introduced a 7 × 7-sized convolution kernel into the convolution block for modeling long-distance pixel-to-pixel dependencies. Moreover, the CBAM guides the network to learn the weights between various channels, which enhances the fitting ability of MobileViT to image feature representation.
- We conducted comparative experiments on several datasets obtained under different scenarios, and the results revealed that our method not only competes with similarly sized lightweight networks but also outperforms state-of-the-art heavyweight networks.

## 2 Materials and methods

### 2.1 Datasets

**Figure 1:**
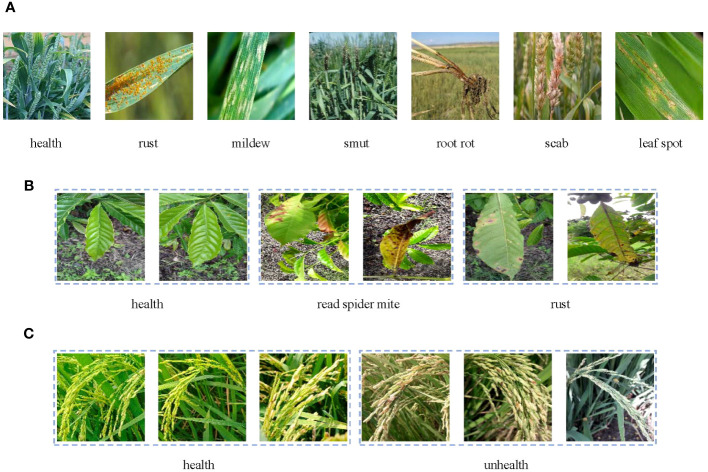
Sample images from the **(A)** wheat dataset, **(B)** coffee dataset, and **(C)** rice dataset.

We randomly divided three datasets into a training set, validation set, and testing set according to the ratio of 8:1:1. **Table 1** shows the details of each dataset and how many samples comprised each subset. **Figure 1** displays some samples of the datasets.

**Table 1:** Data distributions for the datasets used in our comparative experiments.

Name	Class	Diseases	Training set size	Validation set size	Testing set size
Wheat	0	health	528	65	59
	1	rust	673	83	77
	2	mildew	282	34	32
	3	smut	674	83	75
	4	root rot	381	46	41
	5	scab	391	48	45
	6	leaf spot	378	47	45
Coffee	0	healthy red	353	43	39
	1	spider mite	136	16	15
	2	rust	324	39	35
Rice	0	healthy	407	50	44
	1	unhealthy	413	50	43

#### 2.1.1 Wheat

The wheat (Lian, 2022) dataset comprises 4087 images of varying sizes depicting seven different categories of wheat diseases. The images include the real-world environmental factors that interfere with identifying the wheat crop, such as sky, soil, and weeds.

#### 2.1.2 Coffee

The coffee (Parraga-Alava et al., 2019) dataset contains three types of coffee leaves: healthy, red spider mite, and rust. Images of the same size and resolution are included in each category of leaves. The dataset was collected in a natural field environment, where the background of the pictures contains various disturbances such as weeds and soil. Since some sample features are not significant enough, we selected a thousand of them to build a new dataset.

#### 2.1.3 Rice

The rice (Sethy, 2020) dataset lends itself to the classical binary classification problem as it contains samples classified simply as either healthy or unhealthy rice. The resolution of the images in this dataset varies in size. Furthermore, some of the images in this dataset have a uniform white background, which makes the dataset ideal for testing model performance in both a controlled laboratory environment and a real field environment.

### 2.2 Our proposed method

#### 2.2.1 Overall structure of PMVT

**Figure 2:**
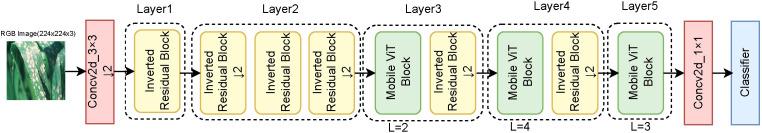
Overview of the PMVT model. ↓2 means to downsample the feature map twice, and L stands for repeated stacking of L MobileViT blocks. For computer vision classification tasks, we use a classifier composed of an average pooling layer and a fully connected layer.

**Figure 2** depicts the overall structure of our model, which comprises five layers. Before pushing input into the block, the feature map is downsampled using a 3 × 3 convolution; this is followed by an inverted residual block or a standard transformer encoder. The inverted residual block is used to extract local features of the image and capture the long-distance dependencies between distant pixels. The MobileViT block uses a self-attention mechanism to model the global relationship of the image and employs a CBAM block to make up the channel attention and spatial attention information. The channel dimension is expanded by four times using a 1 × 1 convolution in the last layer of the network to better adapt to computer classification tasks. PMVT contains three different network sizes: extra extra small (XXS); extra small (XS); and small (S)). These sizes correspond to those in MobileViT.

#### 2.2.2 Inverted residual block

**Figure 3:**
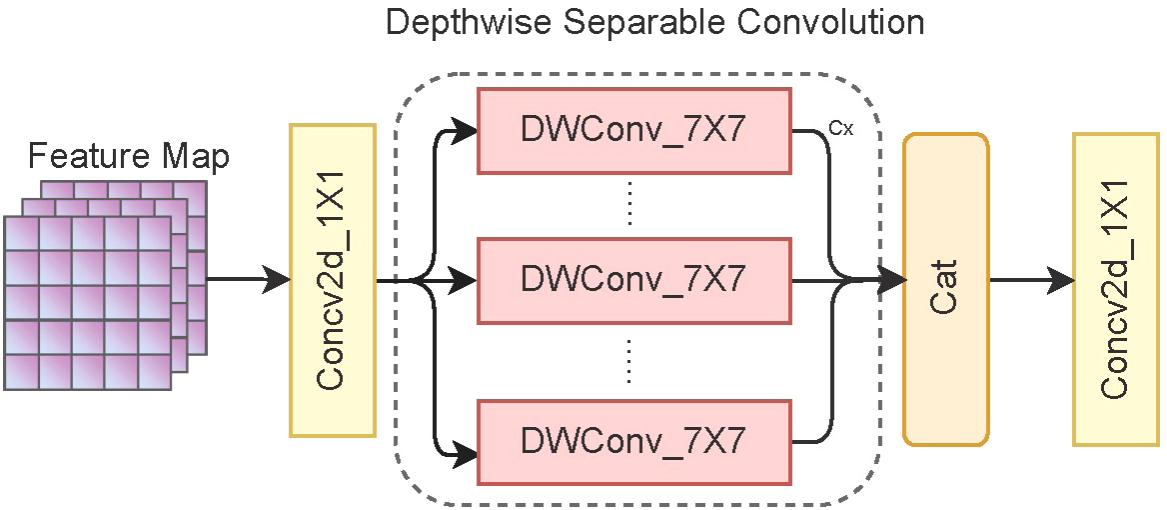
Structure of the inverted residual block. C_×_ represents the feature information obtained by convolving each channel of the feature map using a convolution kernel.

An inverted residual block is a standard convolutional structure comprising three convolution kernels. Before extracting image features, a 1 × 1 convolution kernel is used to increase the channel dimension, generally by two times. Then, we replace the 3 × 3 convolution kernel of the original MobileViT with a 7 × 7 convolution kernel, thus making it easier to capture long-distance dependencies between pixels. In addition, depthwise separable convolutions are used to reduce the computational complexity of the model and increase the inference speed. Finally, we use a 1 × 1 convolution kernel to restore the channel dimension of the image. **Figure 3** shows the overall structure of the inverted residual block.

#### 2.2.3 Mobile ViT block

**Figure 4:**
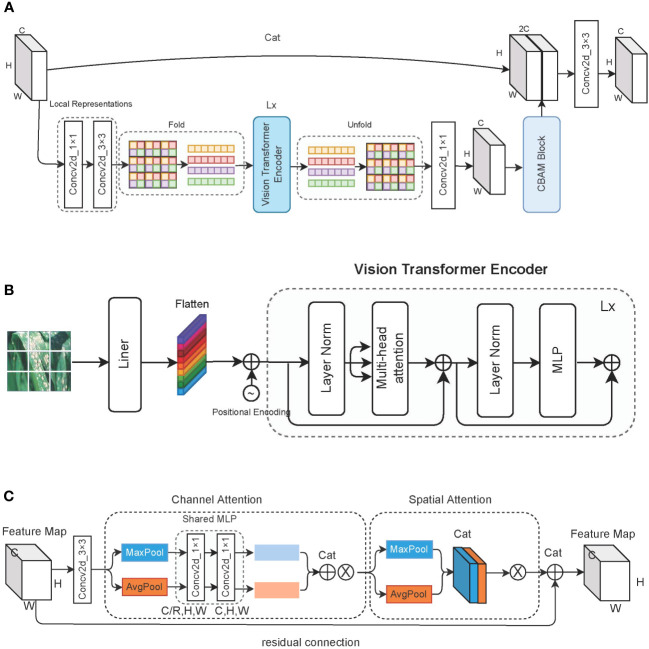
Detailed description of the vision transformer block. **(A)** The overall structure of the vision transformer block; **(B)** the structure of the vision transformer block encoder; and **(C)** the architecture of the CBAM block, where ⊗ represents the multiplication with the original feature map.

As described in **Figure 4A**, learning global representations of feature maps using 1 × 1 and 3 × 3 convolutions. Before entering the standard transformer encoder, the same color patch at the same position is taken out and put into the same sequence for self-attention calculation. This measure allows us to learn the global representation information of the image in a more blocky manner and reduce the computational cost of the self-attention mechanism. Through the 1 × 1 convolution kernel, the output of the transformer is restored to the original channel dimension, and the channel attention and spatial attention information are learned through the CBAM block. Finally, the obtained feature map is spliced with the original feature map to prevent loss of feature information and is then input to the next stage after a 3 × 3 convolution.

#### 2.2.4 Vision transformer encoder

As shown in **Figure 4B**, the encoder used to learn image features consisting of standard transformer blocks. First, an image with dimensions [C*,H,W*] is divided into patches of P size, and a linear transformation is applied to each patch for flattening. Positional encoding information is then applied to each patch; through this, each patch then has dimensions of $\left[\frac{H}{P_{i}},\frac{W}{P_{i}},C\right]$ . Next, we use three learnable parameter matrices to multiply each patch to get queries(*W^Q^*), keys (*W^K^*), and values (*W^V^*). For patch i, we apply the dot product to the query matrix with the key matrix of the remaining patches, and then we divide by the number of key matrix elements. Finally, we apply the softmax function to obtain the attention scores of the remaining patches for patch i. These attention scores are multiplied by the value matrix of patch i to obtain the feature information. Equation 1 illustrates the process of the entire attention mechanism. MLP comprises two fully connected layers and employs an incentive compression mechanism to learn interaction information between different dimensions.


(1) $$self-attention=soft\max\left(\frac{QK^{T}}{\sqrt{d_{k}}}\right)\;\times \;V$$

#### 2.2.5 CBAM block

The CBAM block is composed of a channel attention module and a spatial attention module, and it uses a 3 × 3 convolution kernel to preprocess the feature map before insertion. We pass the input feature map through a parallel average pooling layer and max pooling layer, and then we change the feature map from [C*,H,W*] to [C,1,1] dimensions. The shared MLP module comprises two 1 × 1 convolution kernels, which compress the number of channels to R times the original number and then expand it back to the original number of channels. The feature maps obtained by the average pooling layer and the max pooling layer are spliced to obtain the weights of each channel, which are finally multiplied by the original feature map. Equation 2 describes the weight assignment process of the channel attention module. *σ* stands for using Sigmoid as the activation function, W_1_ ∈ ℝ*^C/r^* ^×^ *^C^*, and W_1_ ∈ ℝ*^C/r^* ^×^ *^C^*. W_1_ and W_0_ are shared weights for the two inputs of the max pooling layer and the average pooling layer.


(2) $$\begin{array}{c} M_{c}\left(F\right)=\sigma \left(MLP\left(AvgPool\left(F\right)\right)+MLP\left(MaxPool\left(F\right)\right)\right)\\ =\sigma \left(W_{1}\left(W_{0}\left(F_{avg}^{c}\right)\right)+W_{1}\left(W_{0}\left(F_{max}^{c}\right)\right)\right)\; \end{array}$$

The output of the channel attention module is obtained through the max pooling layer and average pooling layer. We acquire two feature maps with dimensions of [1*,H,W*], and then we splice them. Through a 7 × 7 convolution, we obtain a feature map of one channel and multiply it by the original feature map. Equation 3 shows the forward process of the spatial attention module, while **Figure 4C** shows the forward process of the entire CBAM block.


(3) $$\begin{array}{c} M_{s}\left(F\right)=\sigma \left(f^{7\times 7}\left(\left[AvgPool\left(F\right);MaxPool\left(F\right)\right]\right)\right)\\ =\sigma \left(f^{7\times 7}\left(\left[F_{avg}^{s};F_{max}^{s}\right]\right)\right)\; \end{array}$$

### 2.3 App for plant disease identification

**Figure 5:**
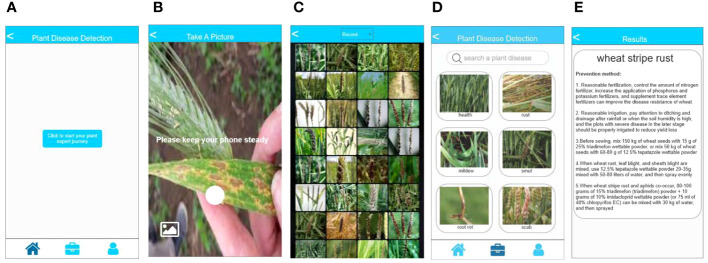
Introduction of plant disease identification app. **(A)** the main page of the app; **(B)** the page for camera recognition; **(C)** the page to select local albums for recognition; **(D)** the page for disease search; and **(E)** the page displaying disease identification results.

We export the trained model to an open neural network exchange (ONNX) file format to preserve crucial details such as structure and weights. The model is converted into an NCNN file format for storage to facilitate deployment on a mobile terminal for inference because the NCNN format is a high-performance neural network inference framework optimized for mobile platforms. Subsequently, the structure and weight information of the model are extracted for plant disease identification using the C++ language. The XML language is used to define the layout and appearance of the application front-end interface. Lastly, the back-end interaction of the application is developed using the JAVA language, while the MySQL database is used for storing plant diseases and related information. As shown in **Figure 5**, the app possesses the capability to perform photo identification using the camera of the device (**Figure 5B**). Alternatively, it allows users to select pictures from their album for identification (**Figure 5C**). Furthermore, users have the option to search for plant diseases based on specific conditions or criteria (**Figure 5D**). The application then presents the relevant categories of plant diseases based on the selected pictures or conditions. **Figure 5E** displays the final identification results of plant diseases and the corresponding control methods.

### 2.4 Experimental details

Data augmentation has been shown to improve model robustness and generalization. Before training the network, all images are uniformly resized to 224 × 224. The samples in the training, validation, and test sets are randomly rotated and cropped along the center. Finally, we normalize all images using standard deviation and mean square deviation. **Table 2** describes our hyperparameter settings for model training.

**Table 2:** Hyperparameter settings for training.

Name	Value	Description
Epochs	100	Number of times the model was trained
Batch size	32	Number of samples selected for one training
Optimizer	AdamW	Tool used to bootstrap network update parameters
Learning rate	0.0001	Tunes parameters in optimization algorithms
Loss function	Cross Entropy	Evaluates the gap between the predicted value and the true value

### 2.5 Model evaluation

In this study, we use top-1 accuracy (Equation 4) to determine the highest accuracy that the model can achieve. We also use precision (Equation 5) and recall (Equation 6) to evaluate the performance of the model. Parameters, floating point operations per second (FLOPs), and frames per second (FPS; the number of images the model processes per second) are used to express the inference speed of the model. True positive (TP) means that the predicted positive sample is actually a positive sample; false positive (FP) indicates that the predicted positive sample is actually a negative sample; true negative (TN) means that the predicted negative sample is actually a negative sample; and false negative (FN) means that the predicted negative sample is actually a positive sample.


(4) $$Top-1\;Accuracy=\frac{TP+TN}{TP+TN+EP+FN}$$

(5) $$\text{Precision}=\frac{TP}{TP+FP}$$

(6) $$\text{Recall}=\frac{TP}{TP+FN}$$

### 2.6 Experimental setup

All experiments run on a deep learning–based cloud platform. The hardware configuration is a 14-Core VV Intel(R) Xeon(R) Gold 6330 CPU @ 2.00 GHz, with 45 GB of RAM and an NVIDIA GeForce RTX 3090 GPU. The operating system is Ubuntu 18.04, and PyTorch 1.9.0 and Python 3.8 are used as software support.

## 3 Results and conclusions

### 3.1 Results

We selected several typically used CNN-based and ViT-based networks for comparison with our model. These include lightweight networks such as SqueezeNet (Iandola et al., 2016), ShuffleNetV2 (Ma et al., 2018), MobileNetV3 (Howard et al., 2019), MobileFormer (Chen et al., 2022), EfficientNet (Tan and Le, 2019), and Deit (Touvron et al., 2021) models. We also chose many heavyweight networks such as PoolFormer (Yu et al., 2022b), CVT (Wu et al., 2021), TNT (Han et al., 2021), and ResNet (He et al., 2016) for comparison. Additionally, we chose a wheat dataset with multiple components (such as roots, stems, and leaves) to evaluate model performance on images depicting diverse conditions. The coffee dataset was employed to assess the performance of our method when confronted with complex backgrounds. Moreover, the rice dataset was used to investigate the classical binary classification problem.

**Figure 6:**
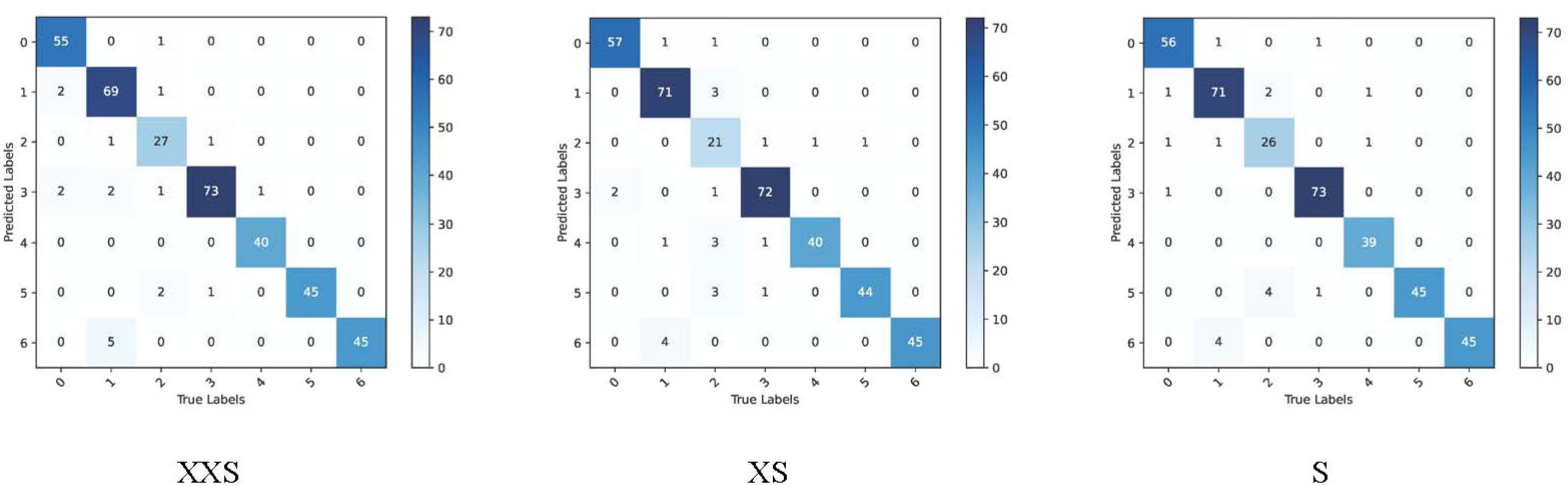
Confusion matrix of the PMVT model on the wheat dataset.

**Figure 7:**
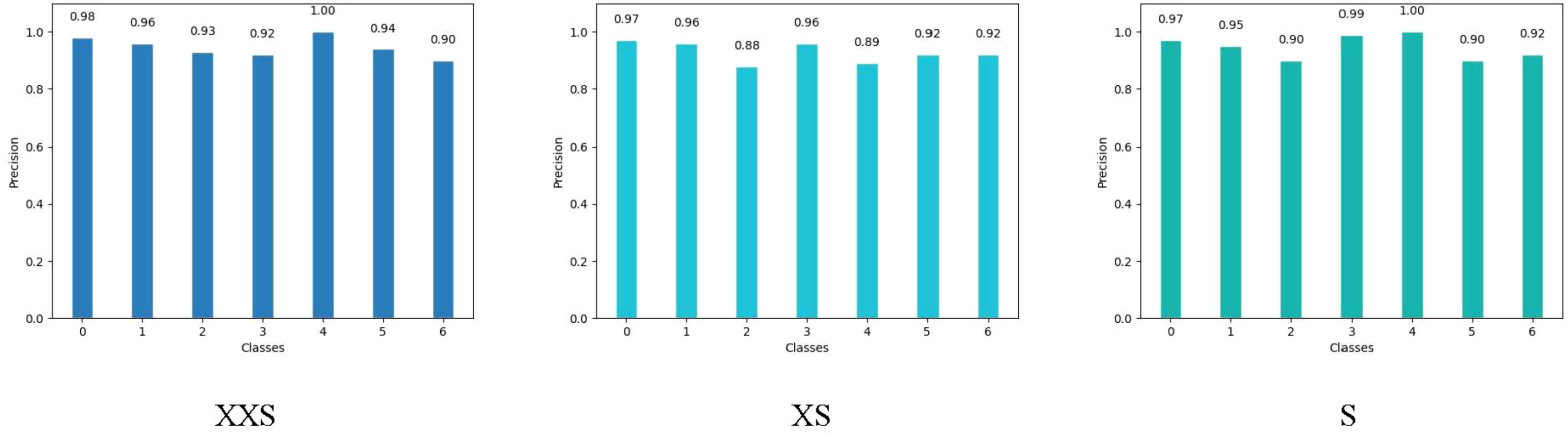
Precision of the PMVT model on the wheat dataset.

**Figure 8:**
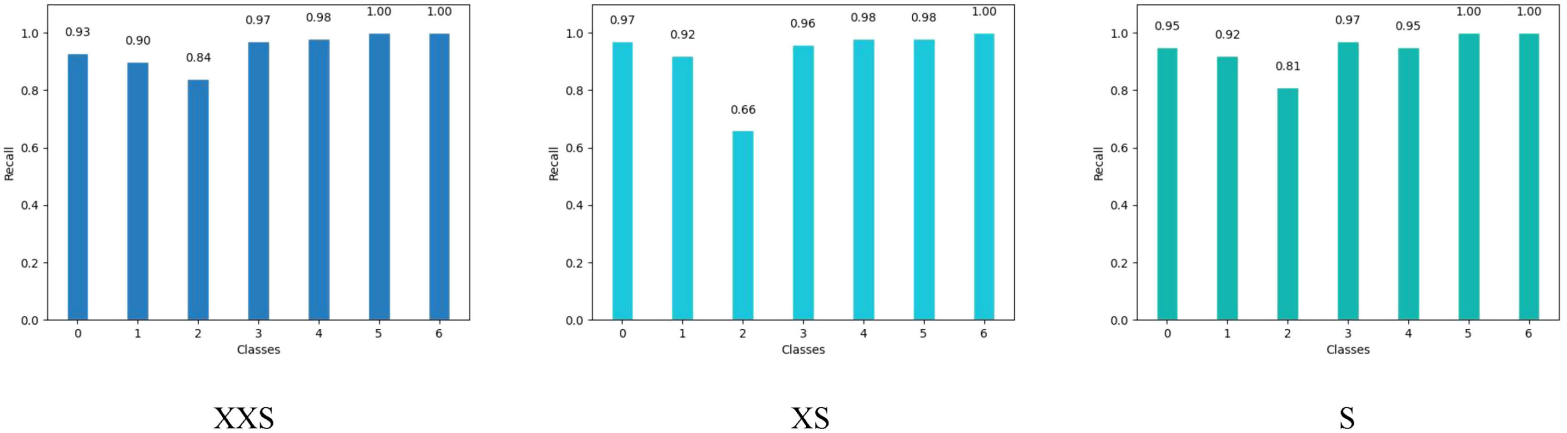
Recall of the PMVT model on the wheat dataset.

We chose the wheat dataset to verify the generalizability of PMVT under a real crop growth cycle. We can see from **Table 3** that our proposed network achieved the best top-1 accuracy when compared with networks with similar parameters. Among the lightweight networks, MobileNetV3 achieved an accuracy rate of 92.0%, whereas EfficientNet-B0 achieved a higher accuracy rate of 94.1%. Our PMVT reached state-of-the-art accuracy with rates of 93.6 and 94.7, respectively. In comparing heavyweight networks, the PMVT model achieved an accuracy rate of 94.9% using only 5.06 M parameters, outperforming ResNet-101, which achieved an accuracy of 94.1% but used 42.5 M parameters. This proves that the proposed model is effective compared to the original MobileViT. **Figure 6** presents the confusion matrix of our proposed model. **Figure 7** depicts the precision of the PMVT model, while **Figure 8** illustrates its recall.

**Table 3:** Comparison of the PMVT model with other backbone models on three datasets (the FPS indicator is calculated on the desktop computer, and bold text highlights the best-performing network).

Methods	Top-1 Accuracy(%)			Parameters (M)	FLOPs (G)	FPS (img/s)
	Wheat	Coffee	Rice			
SqueezeNet-1.0	70.0	79.7	86.2	0.74	0.73	293.0
SqueezeNet-1.1	86.1	83.1	85.1	0.73	0.26	**311.5**
ShuffleNetV2-1.0	89.6	68.5	82.7	1.27	0.15	151.9
MobileNetV3-Small	92.0	66.3	89.7	1.54	**0.06**	170.2
PMVT-XXS (ours)	**93.6**	**85.4**	**93.1**	0.98	0.31	88.5
ShuffleNetV2-1.5	92.5	73.0	86.2	2.50	0.31	148.4
MobileFormer-26M	91.4	77.5	90.8	2.22	**0.03**	53.1
MobileFormer-52M	92.8	79.2	83.9	2.46	0.05	60.7
MobileFormer-96M	92.8	84.2	87.3	3.33	0.09	58.8
MobileNetV3-Large	92.8	72.0	91.9	4.22	0.23	**141.0**
EfficientNet-B0	94.1	84.2	88.5	4.03	0.41	109.9
PMVT-XS (ours)	**94.7**	**86.5**	**97.7**	2.01	0.85	85.3
ShuffleNetV2-2.0	93.6	70.0	91.4	5.38	0.60	146.2
MobileFormer-151M	94.4	75.3	88.5	6.34	**0.10**	42.3
EfficientNet-B1	94.4	79.8	90.8	6.53	0.61	75.3
EfficientNet-B2	93.3	83.1	87.3	7.72	0.70	76.6
Deit-Tiny	91.4	78.7	84.0	5.49	1.08	161.7
PoolFormer-S12	91.4	85.4	85.1	11.39	1.81	**178.3**
CVT-Tiny	93.6	82.0	86.2	19.63	4.08	62.2
TNT-Small	92.8	80.9	88.5	23.40	4.85	67.3
ResNet50	93.9	70.8	90.8	23.53	4.13	125.1
ResNet101	94.1	63.0	88.5	42.50	7.86	66.3
PMVT-S (ours)	**94.9**	**87.6**	**92.0**	**5.06**	1.59	81.3

**Figure 9:**
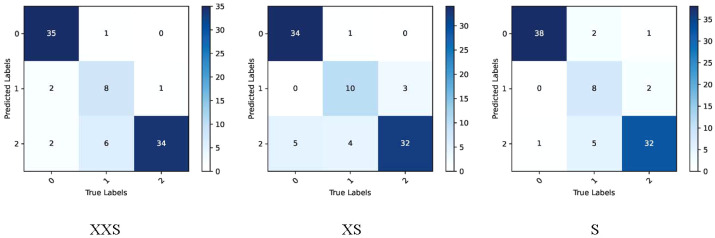
Confusion matrix of the PMVT model on the coffee dataset.

**Figure 10:**
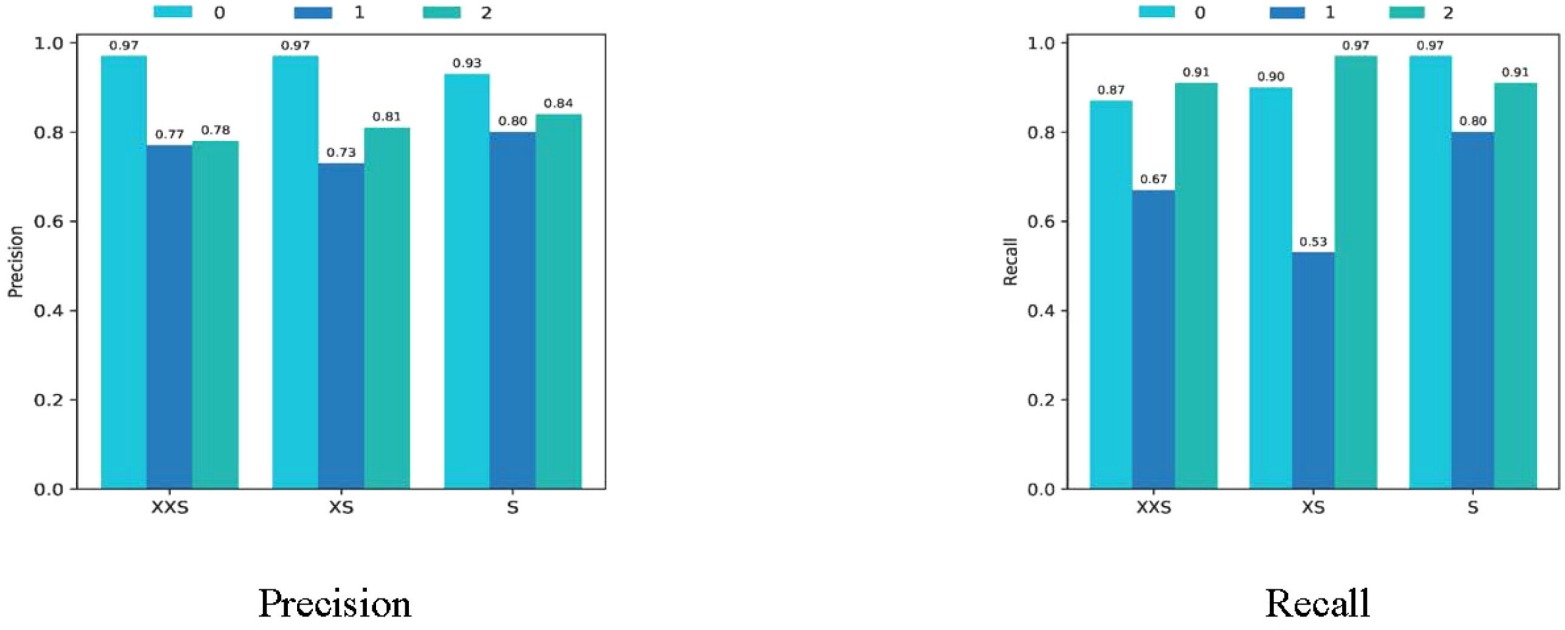
Precision and recall of the PMVT model on the coffee dataset.

The coffee dataset was used to compare the performance of the PMVT models in the field environment. As can be seen from **Table 3**, the traditional lightweight networks did not achieve acceptable accuracy rates. The XXS version of the PMVT model achieved a top-1 accuracy rate of 85.4%, which was 3.5% higher than that of the SqueezeNet-1.1 model. Compared with the MobileFormer-96M model, the XS version of the PMVT model improved accuracy by 2.3% to reach 86.5%. Finally, the S version of the PMVT model achieved an accuracy rate of 87.6% on this dataset; this was an improvement of 2.2% over that obtained by the PoolFormer-S12 model. **Figures 9** and **10** present the confusion matrix, precision, and recall of the PMVT model. It can be seen from the figures that our model does not achieve satisfactory results in identifying red spider mite diseases.

**Figure 11:**
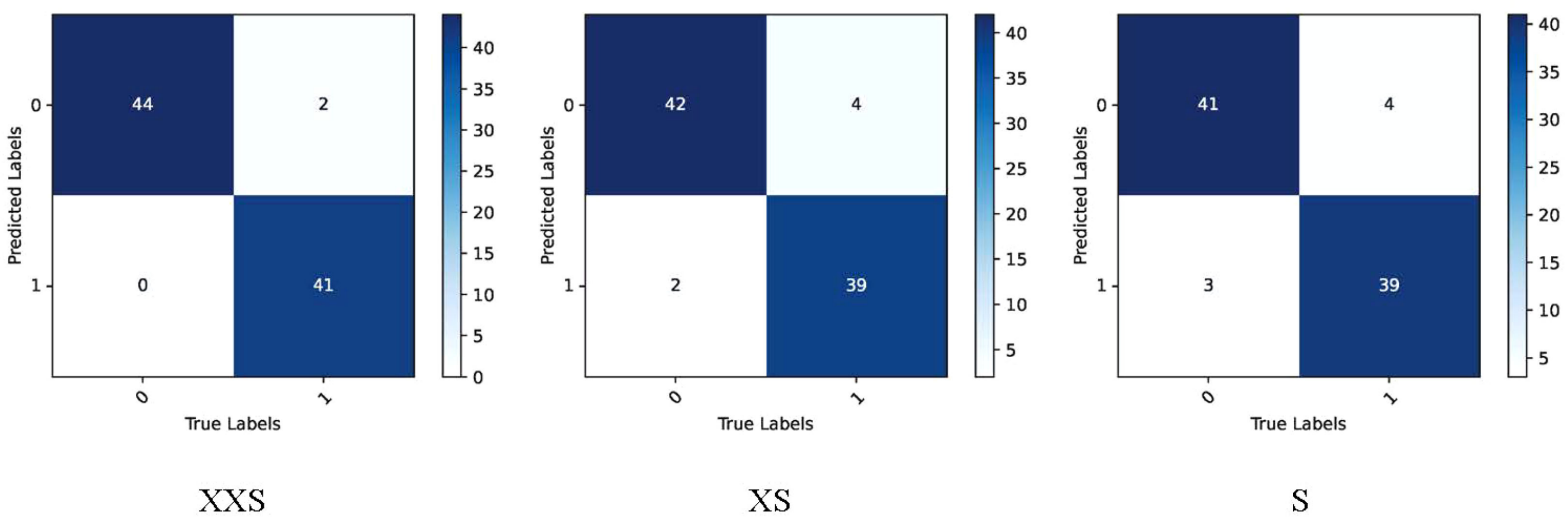
Confusion matrix of the PMVT model on the rice dataset.

**Figure 12:**
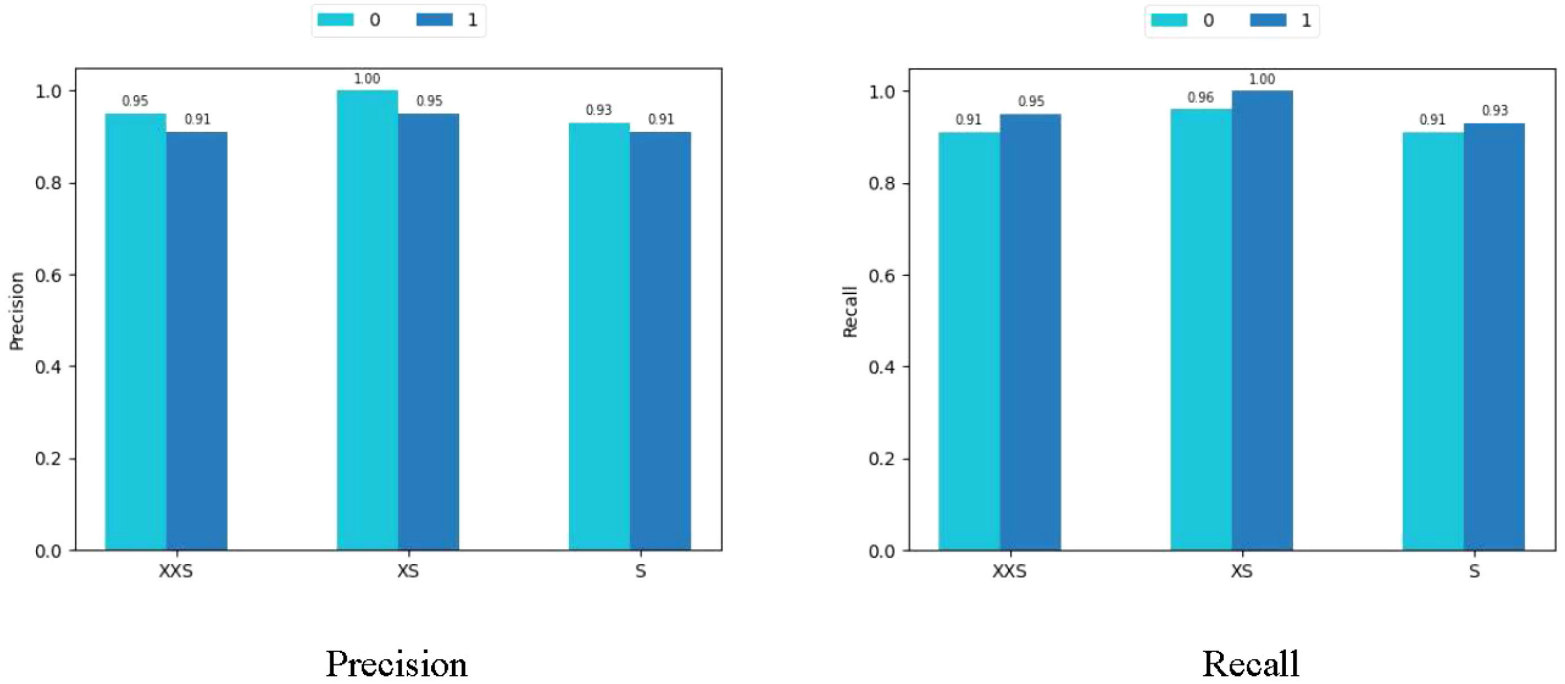
Precision and recall of the PMVT model on the rice dataset.

We applied the rice dataset to simultaneously testing the fitting ability of the PMVT model in a controlled laboratory environment and in a real natural condition. Surprisingly, the XS version of PMVT achieved 97.7% accuracy on this dataset, which was 5.8% higher than the second-highest accuracy (obtained by the MobileNetV3-large model). In addition, the XXS version attained an accuracy of 93.1%, which was 3.4% higher than the baseline of the MobileNetV3-small model. The S version of the PMVT model performed the worst, with an accuracy of 92%; however, it still outperformed the ShuffleNetV2-2.0 model with similar parameters by 0.6%. Upon comparing models with similar sizes, we found that the PMVT model has achieved the best accuracy rate. This proved that our model is very competitive on the classic binary classification problem. **Figures 11** and **12** depict the confusion matrix, precision, and recall of the PMVT model on the rice dataset.

As seen in **Table 3**, our method does not excel in terms of FPS and FLOPs metrics. This because the self-attention mechanism computes the weights between image patches, resulting in numerous matrix calculations and multiplication operations during inference. Consequently, this increases the computational time. Additionally, because of the current immaturity of deep learning framework technology, numerous attention-weight matrices must be stored and processed, thereby occupying a significant amount of memory. Nevertheless, PMVT achieves the best accuracy with only 0.98M parameters. This makes it low-cost and high-accuracy for plant disease identification. As artificial intelligence technology advances, ViT can be better applied to the visual task of plant disease identification.

### 3.2 Ablation studies

The data given in **Table 4**, it demonstrates the effectiveness of each module in our models. +Conv7 × 7 represents using a convolution kernel of size 7 instead of the 3 × 3 convolution in the CNN block based on the MobileViT model. +CBAM uses channel attention and spatial attention integrated in the ViT block based on the MobileViT model. PMVT represents a new backbone network built on the basis of MobileViT using both 7 × 7 convolution kernels and CBAM modules. It can be seen that each component can improve the accuracy of the model to varying degrees.

**Table 4:** Ablation experiments investigating each component in the PMVT model (bold text highlights the best-performing network).

Methods	Wheat(%)	Coffee(%)	Rice(%)	Params(M)	FLOPs(G)
MobileViT-XXS	91.4	83.1	92.0	0.96	0.27
+Conv7x7	92.2(+0.8)	84.0(+1.1)	92.8(+0.8)	0.97(+0.01)	0.30(+0.03)
+CBAM	92.5(+1.1)	84.1(+1.0)	92.6(+0.6)	0.97(+0.01)	0.27
PMVT-XXS	**93.6(+2.2)**	**85.3(+2.1)**	**93.1(+1.1)**	**0.98(+0.02)**	**0.31(+0.04)**
MobileViT-XS	93.3	84.2	94.2	1.94	0.74
+Conv7x7	93.9(+0.6)	85.3(+1.1)	95.8(+1.6)	1.99(+0.05)	0.84(+0.1)
+CBAM	93.6(+0.3)	85.6(+1.4)	96.5(+2.3)	1.95(+0.01)	0.76(+0.02)
PMVT-XS	**94.7(+1.4)**	**86.5(+2.3)**	**97.7(+3.5)**	**2.01(+0.07)**	**0.85(+0.11)**
MobileViT-S	93.9	84.3	89.7	4.95	1.46
+Conv7x7	94.4(+0.5)	85.4(+1.1)	90.9(+1.2)	5.02(+0.07)	1.59(+0.13)
+CBAM	94.4(+0.5)	84.7(+1.4)	91.1(+1.4)	4.98(+0.03)	1.47(+0.01)
PMVT-S	**94.9(+1.0)**	**87.6(+3.3)**	**92.0(+2.3)**	**5.06(+0.11)**	**1.59(+0.13)**

### 3.3 Conclusion

In this paper, we constructed a computationally efficient vision transformer (ViT) model, referred to as PMVT, for the identification of plant diseases. Furthermore, larger convolution kernels and CBAM modules enhanced the model’s feature extraction capability. Comparative experiments were conducted on multiple datasets containing images of plant diseases, thus demonstrating that PMVT outperforms both lightweight and heavyweight networks. Additionally, PMVT outperforms both lightweight and heavyweight networks. PMVT has more powerful generalization capabilities and can be deployed on mobile devices for diagnosing plant diseases in field environments. However, due to the shorter development time of ViT, lightweight ViT models are comparatively slower than traditional lightweight CNNs when processing images. The advancement of deep learning framework technology enables ViT to perform computer vision tasks more effectively.

## Data availability statement

The original contributions presented in the study are included in the article/supplementary material. Further inquiries can be directed to the corresponding author.

## Author contributions

GL: Conceptualization, Methodology, Writing – review & editing. YW: Software, Visualization, Writing – original draft. QZ: Data curation, Validation, Writing – review & editing. PY: Resources, Writing – review & editing. BC: Funding acquisition, Supervision, Writing – review & editing.
